# Chronic stress-induced downregulation of MFN1 contributes to fatty liver in chickens

**DOI:** 10.3389/fvets.2025.1646921

**Published:** 2025-09-25

**Authors:** Ke Zhang, Hui Yu, Huimin Chen, Ziting Guan, Xiaoyu Wang, Jie Liu, Mindie Zhao, Ruqian Zhao, Dancheng Yang, Lei Wu

**Affiliations:** ^1^Key Laboratory of Animal Physiology and Biochemistry, College of Veterinary Medicine, Nanjing Agricultural University, Nanjing, Jiangsu, China; ^2^Department of Nutritional Sciences, Oklahoma State University, Stillwater, OK, United States; ^3^MOE Joint International Research Laboratory of Animal Health and Food Safety, Nanjing Agricultural University, Nanjing, Jiangsu, China

**Keywords:** chronic stress, glucocorticoid, mitochondrial fusion, mitofusin 1, glucocorticoid receptor

## Abstract

**Background:**

Chronic stress is a major contributor to Fatty Liver Syndrome (FLS) in fast-growing broilers, leading to physiological dysfunctions that compromise growth and immune response. This study aimed to investigate the effects of chronic stress on hepatic lipid metabolism and mitochondrial dynamics in broilers.

**Method:**

Forty 1-day-old male AA broilers were randomly allocated into two groups (*n* = 20): control (CON) and corticosterone-treated (CORT). From day 38, the CORT group received twice-daily subcutaneous injections of CORT (4 mg/kg/day) for 7 days to simulate *in vivo* chronic stress model. The loss-of-function approaches in cell culture models were also applied to investigate the role of MFN1 in CORT-induced mitochondrial dysfunction.

**Results:**

Chronic CORT treatment induced significant hepatic steatosis and liver injury in broilers. Furthermore, CORT disrupted mitochondrial function, as indicated by excessive mitochondrial fragmentation, a pronounced decrease in mitochondrial membrane potential (MMP), and aberrant oxidative stress responses in both *in vivo* and *in vitro* models. Studies showed that glucocorticoid receptor (GR)-mediated downregulation of mitofusin 1 (MFN1) plays a critical role in CORT-induced disruption of lipid metabolism. Importantly, restoration of MFN1 expression effectively rescued mitochondrial morphology and function and attenuated lipid accumulation in hepatocytes.

**Conclusion:**

This study reveals a key mechanism by which chronic stress impairs mitochondrial fusion via GR-mediated suppression of MFN1, driving fatty liver development in broilers. These findings underscore the critical role of MFN1 in mitochondrial dynamics and lipid metabolism, offering novel insights for potential therapeutic strategies against fatty liver disease in poultry.

## Introduction

In modern intensive poultry farming, birds are frequently exposed to various environmental and metabolic stressors—such as fluctuations in diet, temperature, humidity, stocking density, and transportation—that trigger excessive glucocorticoid secretion (1). In poultry, corticosterone (CORT) is the primary active glucocorticoid, and its chronic elevation has been directly linked to significant metabolic disturbances. Notably, prolonged CORT exposure promotes the accumulation of triglycerides (TGs) in the liver, leading to severe hepatic steatosis—a condition that impairs animal health, welfare, and overall production efficiency (2). Similarly, acute administration of synthetic glucocorticoids like dexamethasone has been shown to significantly increase hepatic and serum TG levels in chicken (3). Therefore, there is a growing interest within the field to elucidate the precise molecular mechanisms driving glucocorticoid-induced fatty liver in chicken, with the aim of identifying novel therapeutic targets to mitigate this economically important issue.

Glucocorticoids exert their biological effects by activating their specific receptor, known as the glucocorticoid receptor (GR). Upon binding, the GR-glucocorticoids complex moves to the nucleus and binds to glucocorticoid response elements (GREs), regulating the transcription of target genes—such as those promoting lipogenesis and TG accumulation in the liver (4, 5). Additionally, GR can translocate into mitochondria and bind to GREs on mitochondrial DNA (mtDNA), regulating the expression of mitochondrial genes (6). While short-term exposure may boost mitochondrial function, chronic treatment causes severe impairment, including reduced ATP production, increased ROS, and abnormal mitochondrial structure (7, 8). The above dual regulation allows glucocorticoids to profoundly affect cellular metabolism and energy homeostasis. Therefore, the specific molecular mechanisms by which chronic CORT regulate mitochondrial function in poultry remains unclear.

Mitochondria are widely distributed in the liver, which generating ATP to meet energy demands and participating in fatty acid oxidation and ROS production. Therefore, mitochondrial function is crucial for the development of fatty liver (9, 10). As dynamic organelles, the balance between fusion and fission is key to maintaining mitochondrial morphology, size, number, and physiological function. The proper functioning of mitochondria relies on the balance of mitochondrial dynamics (11). This balance is critical for cellular homeostasis, and any disruption can impair mitochondrial function, leading to metabolic disorders such as fatty liver disease (12), liver cancer (13), and diabetes (14). For example, studies in mammalian models have demonstrated that a high-fat diet downregulates the expression of mitochondrial fusion proteins and promotes pathological fission (15, 16). Nevertheless, the role of these dynamic processes, and their regulation by glucocorticoids, in the specific context of chicken fatty liver disease has not been investigated.

Mitochondrial fusion is regulated by several members of the GTPase superfamily, primarily including the outer membrane fusion proteins MFN1 and mitofusin 2 (MFN2), as well as the inner membrane fusion protein optic Atrophy 1 (OPA1). Notably, MFN1 has a higher GTPase hydrolysis efficiency than MFN2 and is involved not only in outer mitochondrial membrane fusion but also in the regulation of inner mitochondrial membrane fusion through interactions with OPA1 (17, 18). Studies have shown that the knockout of MFN1 in mice reduces mitochondrial fusion efficiency, leading to mitochondrial fragmentation (19). Additionally, a reduction in hepatic MFN1 expression has also been reported in rats with non-alcoholic steatohepatitis (NASH) (20). Impaired mitochondrial fusion is also associated with increased oxidative stress, further exacerbating cellular damage (21). Nevertheless, while this relationship is well-established in mammals, it is yet to be elucidated whether the dysregulation of MFN1-mediated fusion is a key mechanistic contributor to CORT-induced hepatic steatosis in chickens.

Therefore, we hypothesize that chronic CORT-induced hepatic steatosis in poultry is mechanistically linked to the downregulation of MFN1 expression, which disrupts mitochondrial fusion dynamics, culminates in mitochondrial dysfunction (evidenced by loss of membrane potential and oxidative stress), and promotes aberrant lipid accumulation. This study identifies MFN1 as a critical molecular target within the GR signaling pathway that underpins the development of fatty liver in poultry, offering a novel strategic foundation for future therapeutic interventions aimed at improving metabolic health in agricultural animals.

## Methods

### Animal experiments

A total of 40 male AA broilers (1-day-old, 44.5 ± 3.4 g) were obtained from Jiangsu Jinghai Poultry Industry Group Co., Ltd. (Jiangsu Province Poultry Science Research Institute) for the study. The broilers were given ad libitum access to feed and water, and were fed a commercial mixed feed (purchased from New Hope Liuhe). The nutrient composition of the broiler basal diet was as follows: 18% crude protein, 9% crude ash, 5% crude fiber, 0.5% total phosphorus, 1.2% calcium, 0.8% sodium chloride, and 0.9% methionine. The lighting schedule followed a 16-h light/8-h dark cycle daily. For the first 3 days, the ambient temperature within the poultry house was maintained at 35 °C, gradually decreasing by 3 °C per week until reaching 23 °C.

At 38 days of age, the broilers were randomly assigned into two experimental groups: the control (CON) group (*n* = 20) and the CORT group (*n* = 20). The CORT group was subjected to chronic stress by receiving subcutaneous injections of CORT (Aladdin, USA) at a dosage of 4.0 mg/kg/day, divided into two doses administered daily for 7 consecutive days (2). The CON group received an equal volume of alcohol-saline solution as a vehicle control. The feed intake of the birds was recorded daily, and body weight was measured weekly throughout the experimental period. At 45 days of age, the broilers were randomly select 12 chickens from each group for subsequent analysis.

### Determination of plasma biochemical parameters and hepatic TG content

Anticoagulated blood samples were collected, placed on ice, and centrifuged at 2000 g for 15 min to separate plasma. Plasma levels of TG, glucose (GLU), total cholesterol (TCH), ALT and AST were measured using an automatic biochemical analyzer. The corresponding assay kits were purchased from Meikang Technology Co., Ltd. Hepatic TG levels were determined using a TG detection kit obtained from Beijing Pulilai Gene Technology Co., Ltd., following the manufacturer’s protocol.

### Histological analysis of liver tissues

Liver tissues were fixed in 4% paraformaldehyde and subsequently embedded in paraffin. Sections 5 μm in thickness were prepared and subjected to Hematoxylin and Eosin (H&E) staining and Oil Red staining. The stained sections were examined under an optical microscope, and images were captured for further histological analysis of tissue lesions.

### Primary chicken hepatocyte isolation

Primary hepatocytes were isolated from 45-day-old AA broilers using the *in situ* two-step perfusion method with collagenase IV (22). Following a fasting period, broilers were anesthetized, and a catheter was inserted into the portal vein. The liver was fixed in situ, and stepwise perfusion was conducted until the liver became softened. The liver was then excised and transferred to a sterile dish, where blood vessels, fat, and connective tissues were carefully removed. The undigested peripheral portions of the liver were trimmed, and the remaining tissue was minced into small fragments. Hepatocytes were filtered through cell strainers, centrifuged at 50 × g for 3 min, and washed three times with DMEM.

### Cell culture and treat

The AML12 cell line was cultured in DMEM/F12 medium supplemented with 10% fetal bovine serum (NEWZERUM, New Zealand), 1% penicillin–streptomycin, and 1% ITS (Biotian, China) at 37 °C in a humidified atmosphere with 5% CO_2_. When the cell density reached 60%, the cells were treated with 50 μM CORT (Aladdin, USA) and 100 μM palmitic acid (PA) for 24 h. The CON group was treated with an equal concentration of 100 μM PA alone.

Specific MFN1 and GR small-interfering RNA (siRNA) was synthesized by QingKe (Qingdao, China), and the sequences of the MFN1, GR siRNAs were in Supplementary Table S4. These siRNAs were transfected into AML12 using the JetPRIME® transfection reagent (jetPRIME®, Polyplus, China). Scrambled siRNA was used as the negative control (si-N.C.).

The coding sequence (CDS) of mouse MFN1 was retrieved from the NCBI database, and primers were designed using CE Design software. Using cDNA derived from mouse tissues as a template, the MFN1 sequence was cloned and ligated into the pcDNA3.1 vector to construct the MFN1 overexpression plasmid.

### Flow cytometry

AML12 were seeded in 12-well plates at a density of 1 × 10^^6^ cells per well. Following the treatment as outlined in the experimental design, the cells were harvested by trypsinization and washed twice with PBS. Subsequently, the cells were incubated separately with MitoSOX^Red^ (MCE, China) and JC-1 (Biotian, China) working solution following the manufacturer’s protocols. After incubation, cells were centrifuged at 600 g for 5 min at room temperature, supernatant discarded, resuspended in 1 mL PBS, and analyzed by flow cytometry (BD FACSVerse, USA).

### RNA extraction and real-time polymerase chain reaction

Total RNA was isolated from liver tissue and cells using Trizol (Tsingke, China) adhering to the manufacturer’s instructions. Subsequently, 1 μg of RNA was reverse transcribed to cDNA using the Reverse Transcription Master Kit (TransGen, China) according to standard protocol. Primers used in this study were designed using Primer Premier 5.0 software, based on primer sequences sourced from PubMed (Supplementary Table S1). qPCR was performed using the AQ601 kit (TransGen, China) with results normalized to PPIA, which served as the internal control for mRNA quantification. The relative gene expression was calculated using the (2^-∆∆Ct^) method, with PPIA as the reference gene.

### Western blotting

Total proteins from tissues and cells were extracted using the RIPA lysis buffer (1% Triton-100, 0.5% sodium deoxycholate, 150 mmol/L NaCl, 0.1% SDS, 50 mmol/L Tris, along with 1% protease inhibitor and 1% phosphatase inhibitor), and protein concentrations were determined using a BCA protein assay kit (TransGen, China). For analysis, 30 μg of tissue protein or 20 μg of cell protein was separated by SDS-PAGE and then transferred to nitrocellulose (NC) membranes. The membranes were blocked with 5% skim milk and incubated with primary antibodies overnight at 4 °C. Subsequently, the membranes were incubated with secondary antibodies for 2 h at room temperature, and the proteins were detected with an ECL chemiluminescence kit (Biosharp, China) and and visualized using a protein imager (Tannon-5200, China). Band intensity was quantified using ImageJ software (Wayne Rasband, National Institutes of Health). The antibodies employed in this study are listed in Supplementary Table S3.

### Bodipy and Nile Red staining

AML12 were seeded in 24-well plates at a density of 1 × 10^^6^ cells per well. Following the treatment as outlined in the experimental design, the cells were harvested by trypsinization and washed twice with PBS. Subsequently, the cells were incubated separately with BODIPY working solution (Beyotime, China), DAPI (Beyotime, China) and Nile Red staining solution (Solarbio, China) at 37 °C in the dark for 15 min. Then the stained cells were then washed three times with PBS and observed under a fluorescence microscope.

### Chromatin immunoprecipitation assay (ChIP)

After crosslinking and lysing the treated cell samples, the lysate was subjected to sonication to obtain DNA fragments of approximately 300–500 bp. Each sample was divided into positive, negative, and input groups, with each group containing 99 μg of chromatin. For the positive group, an antibody against GR (Proteintech, China) was added and incubated overnight to form the antibody–antigen complex. Subsequently, Protein A/G agarose beads were added to both the positive and negative groups and incubated on a shaking platform at 4 °C for 2 h. The beads were then washed and eluted to isolate the DNA-protein-antibody complex. After reversing the crosslinking and removing RNA and proteins, purified DNA was extracted using the phenol/chloroform method. The released DNA was quantified using real-time PCR, and the enrichment of the target genes was analyzed. The list of primers used in the ChIP analysis is provided in the Supplementary Table S2.

### Statistical analysis

The data obtained from this study were analyzed using appropriate statistical methods to ensure the reliability of the results. All data are presented as means ± standard error of the mean (SEM). Differences between groups were assessed using *t*-tests, with *p*-values less than 0.05 considered statistically significant and *p* < 0.01 indicating a highly significant difference. Data analysis was conducted using GraphPad Prism 8.0 software (GraphPad Software Inc., La Jolla, CA). All experiments were repeated at least three times to confirm the reproducibility of the results.

## Results

### Chronic corticosterone treatment induces lipid accumulation and disrupts mitochondrial integrity in broilers

**Figure 1:**
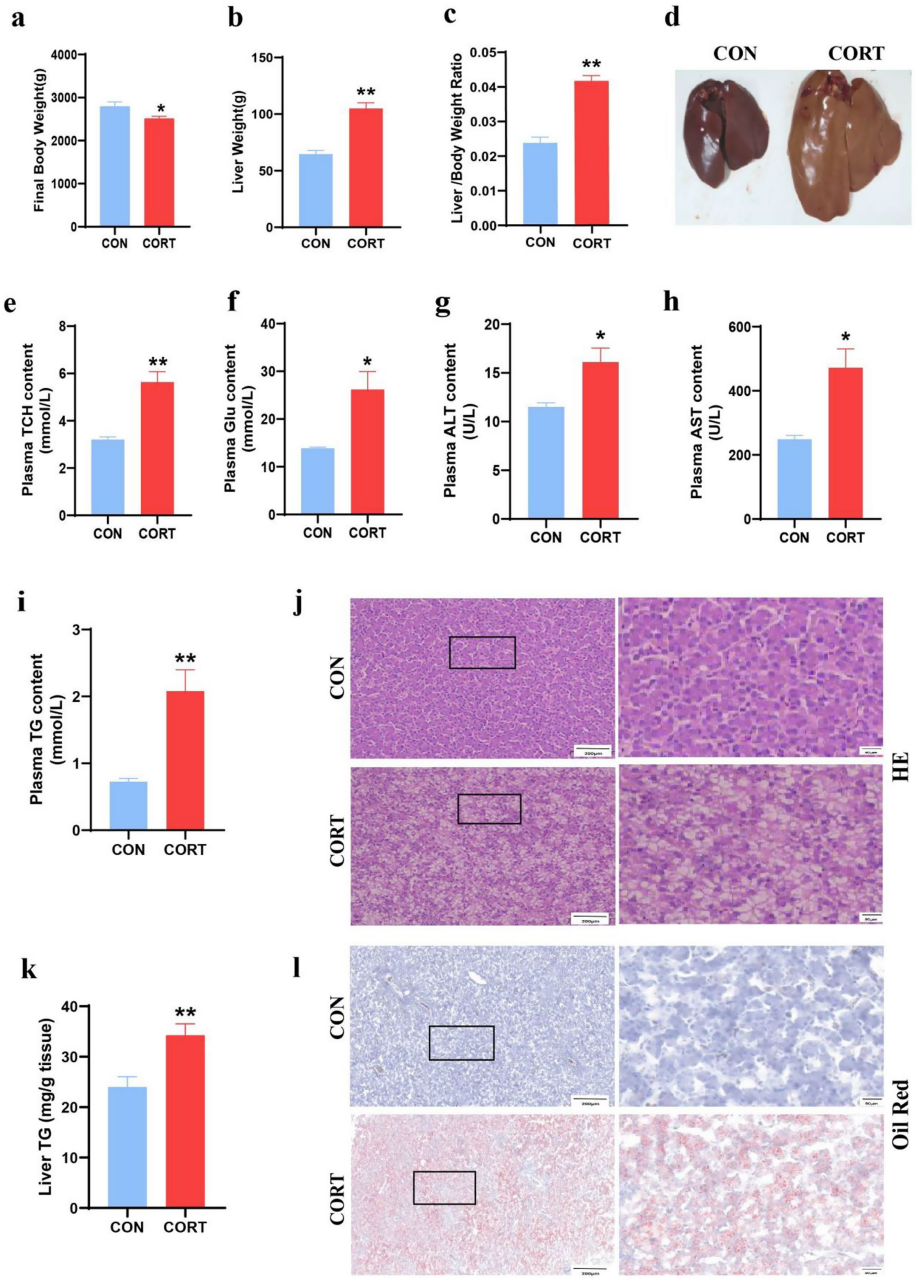
Corticosterone treatment induces abnormal lipid deposition in broilers. **(a)** Final body weight (*n* = 10). **(b)** Liver weight (*n* = 10). **(c)** Liver-to-body weight ratio (*n* = 10). **(d)** Representative liver images. **(e)** Plasma TCH (*n* = 10). **(f)** Plasma Glu (*n* = 10); **(g)** Plasma ALT (*n* = 10). **(h)** Plasma AST (*n* = 10). **(i)** Plasma TG (*n* = 10). **(j)** Representative images of HE staining of liver sections (*n* = 6). **(k)** TG content in liver (*n* = 10). **(l)** Oil Red staining of hepatic lipid (*n* = 6). Values are Mean ± SEM, *p < 0.05, **p < 0.01, compared with the CON group.

**Figure 2:**
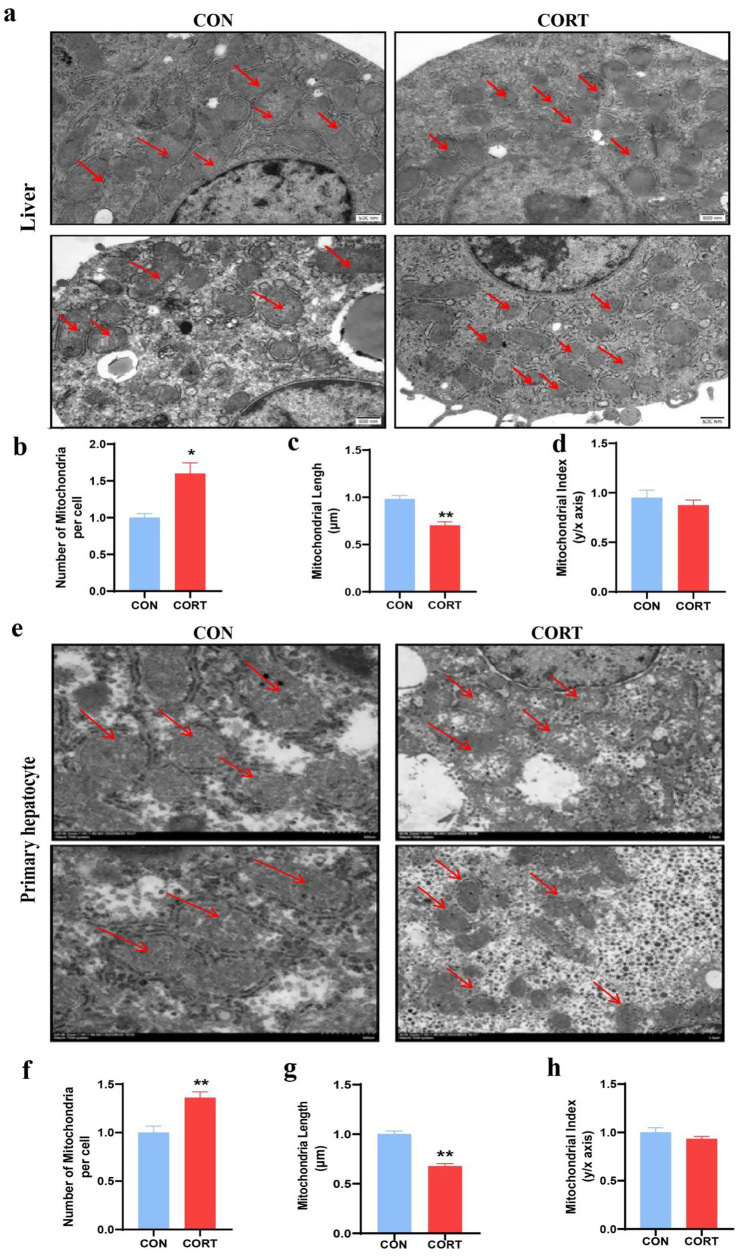
Corticosterone treatment induces abnormal mitochondrial morphology in liver and primary hepatocytes of chicken. **(a)** TEM of liver mitochondria (Scale bars represent 500 nm, *n* = 3). **(b–d)** Changes in mitochondrial number, length and aspect ratio in the liver. **(e)** TEM of primary hepatocyte mitochondria (Scale bars represent 1 μm, *n* = 3). **(f–h)** Changes in mitochondrial number, length and aspect ratio in the primary hepatocytes. Values are Mean ± SEM, *p < 0.05, **p < 0.01, compared with the CON group.

Continuous treatment with CORT for 7 days led to a significant reduction in body weight in broilers (Figure 1a) and pronounced liver enlargement (Figures 1b,c). Also, the liver can be clearly seen to be yellow in the CORT group (Figure 1d). Biochemical analyses indicated substantial increases in plasma TCH, GLU, ALT and AST levels, accompanied by marked elevations in TG (Figures 1e–i). Liver triglyceride levels were also significantly elevated (Figure 1k). HE staining demonstrated CORT treatment-induced hepatocyte swelling and prominent vacuolation of lipid droplets (Figure 1j). Additionally, Oil Red staining of liver sections revealed extensive lipid droplet deposition on the liver surface (Figure 1l). Mitochondria are crucial for the regulation of fatty acid metabolism and synthesis. Transmission Electron Microscopy (TEM) analysis of liver mitochondria (Figure 2a) revealed that CORT treatment resulted in significant mitochondrial fragmentation, along with a marked reduction in mitochondrial diameter (Figure 2c) and a substantial increase in mitochondrial numbers (Figure 2b). Notably, no significant changes were observed of the mitochondrial aspect (Figure 2d). Similar mitochondrial alterations were evident in hepatocytes, as shown by TEM analysis (Figure 2e). The results indicated a significant reduction in the diameter of primary hepatocyte mitochondria (Figure 2g) and an increase in their numbers (Figure 2f), with no notable changes in the mitochondrial aspect (Figure 2h). In summary, CORT treatment resulted in morphologically disordered mitochondria and liver damage, which contributing to the development of fatty liver.

### Corticosterone treatment disrupts mitochondrial dynamics

**Figure 3:**
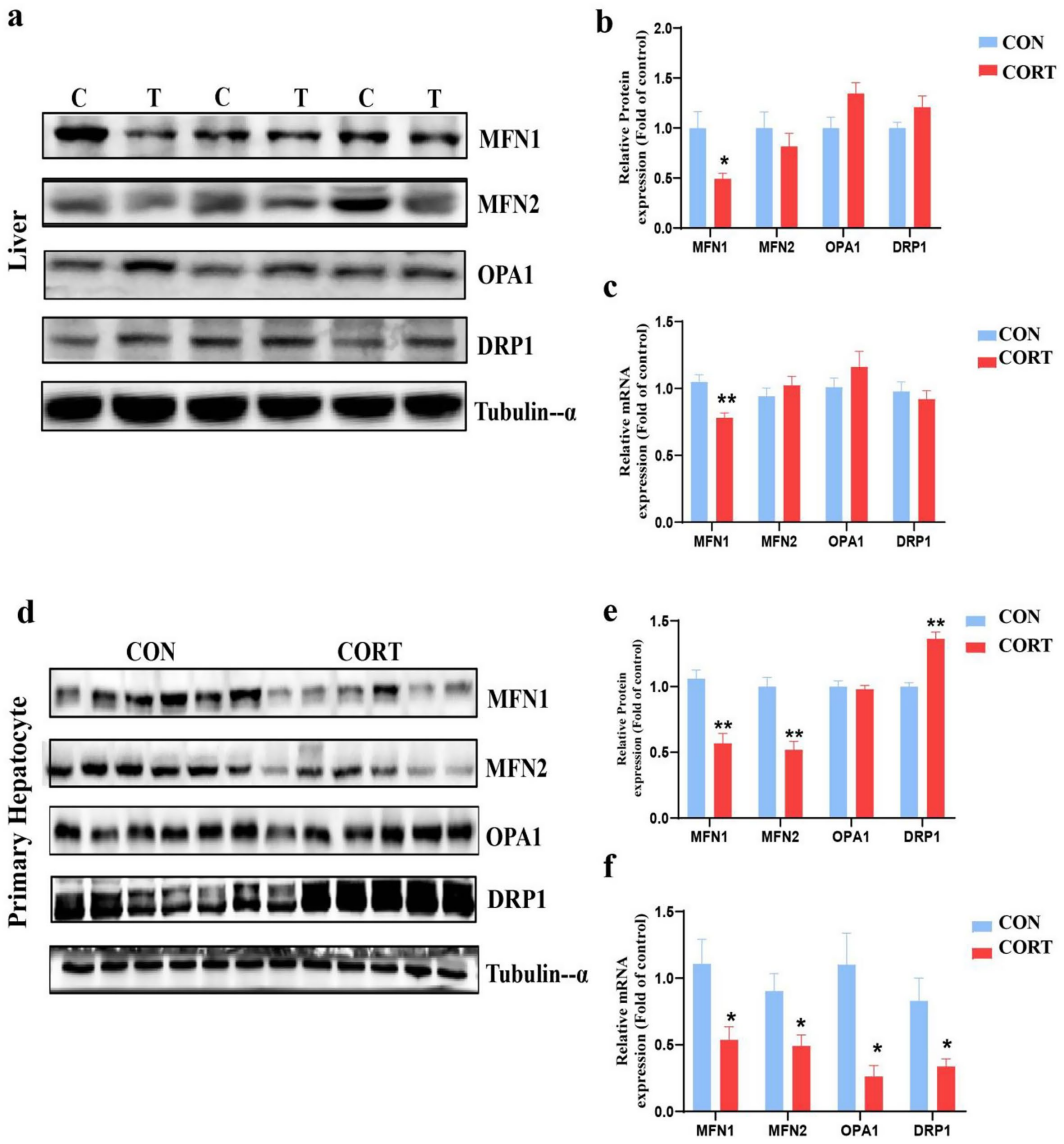
Corticosterone treatment induces imbalance in mitochondrial dynamics in broilers. **(a, b)** Expression of mitochondrial dynamics-related proteins in the liver (*n* = 6). **(c)** mRNA expression of mitochondrial dynamics-related genes in the liver (*n* = 10). **(d,e)** Expression of mitochondrial dynamics-related proteins in primary hepatocytes (*n* = 6). **(f)** mRNA expression of mitochondrial dynamics-related genes in primary hepatocytes (*n* = 6). Values are Mean ± SEM, *p < 0.05, **p < 0.01, compared with the CON group.

Mitochondrial integrity is predominantly influenced by the dynamic balance between fusion and fission processes. Analysis of proteins and genes related to mitochondrial dynamics revealed a significant reduction in the mitochondrial fusion protein MFN1 at both the gene and protein levels in the liver. In contrast, other fusion proteins, including MFN2 and OPA1, as well as the fission protein Dynamin-related protein 1 (DRP1), showed no significant changes (Figures 3a–c). Similarly, in primary hepatocyte mitochondria, MFN1 levels were significantly diminished at both the gene and protein levels, while levels of fusion proteins MFN2 and OPA1 were reduced at the mRNA level. Notably, although protein levels of the fission protein DRP1 increased, its mRNA levels decreased (Figures 3d–f). These findings suggest that the mitochondrial fusion protein MFN1 plays a crucial role in the effects of CORT treatment.

### Corticosterone treatment leads to lipid accumulation, mitochondrial dysfunction in AML12

**Figure 4:**
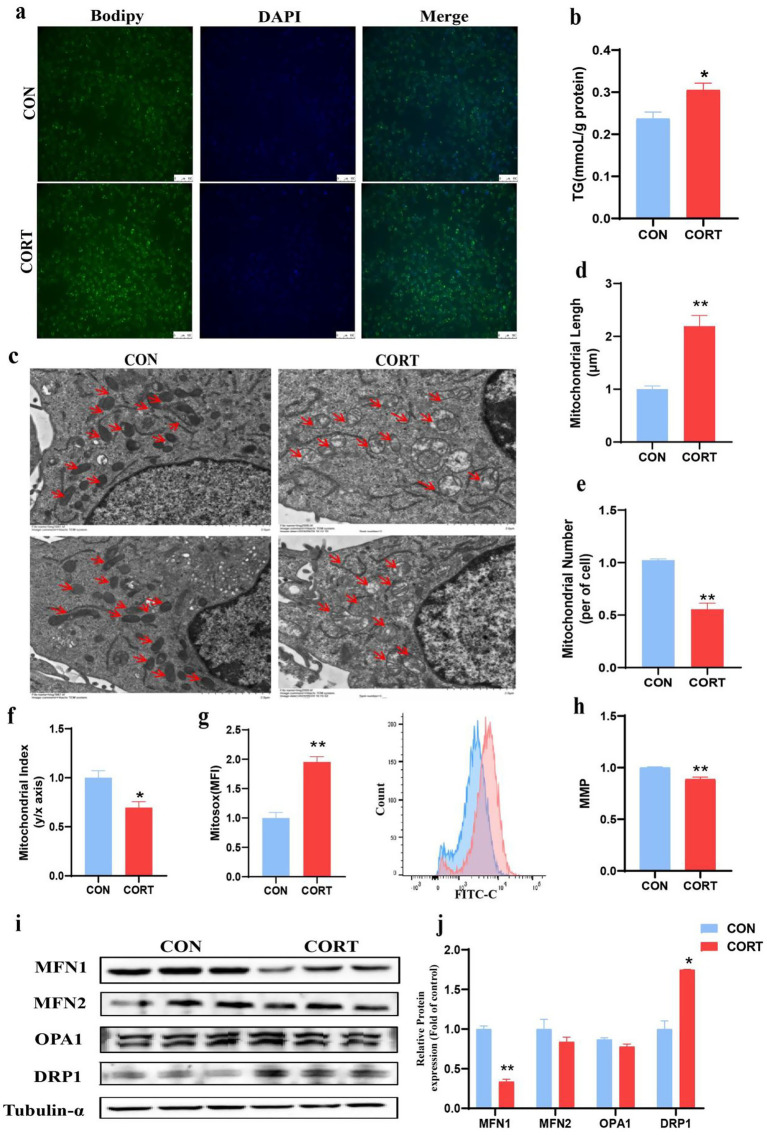
Corticosterone treatment induces lipid deposition and mitochondrial dysfunction in AML12. **(a)** Bodipy 493/503 fluorescence visualized by fluorescence microscopy (scale bars represent 100 μm) (*n* = 3). **(b)** Triglyceride content (*n* = 6). **(c)** TEM images of mitochondria (Scale bars represent 2 μm, *n* = 3). **(d–f)** Changes in mitochondrial number, length and aspect ratio. **(g)** Mitochondrial ROS levels (*n* = 3). **(h)** Mitochondrial membrane potential (*n* = 3). **(i, j)** Expression of mitochondrial dynamics-related proteins. Values are Mean ± SEM, *p < 0.05, **p < 0.01, compared with the CON group.

To investigate the involvement of MFN1 in CORT-induced fatty liver, AML12 were treated with 100 μM CORT combined with 50 μM PA for 24 h for *in vitro* validation. Bodipy staining results demonstrated that CORT exacerbated lipid droplet accumulation in hepatocytes (Figure 4a), and TG levels in AML12 were significantly elevated (Figure 4b). TEM analysis of AML12 mitochondria (Figure 4c) indicated that CORT treatment led to mitochondrial swelling and rupture of cristae, differing from the *in vivo* observations. Specifically, the number of mitochondria was significantly reduced (Figure 4e), while their diameter was significantly elongated (Figure 4d). Both the shape of mitochondria were elongated, resulting in a significant decrease in mitochondrial index compared to the CON group (Figure 4f). Mitochondrial integrity is essential for normal function. Utilizing the MitoSOX^Red^ probe, we found that CORT treatment aggravated mitochondrial oxidative stress, leading to a significant increase in ROS levels (Figure 4g) and a marked decline in MMP (Figure 4h). Further analysis of mitochondrial dynamics-related proteins in AML12 revealed that CORT treatment significantly inhibited the expression of the MFN1 protein (Figures 4i,j). Collectively, these results suggest that CORT-induced dysregulation of MFN1 leads to mitochondrial functional impairment, subsequently promoting the progression of fatty liver.

### Overexpression of MFN1 alleviates corticosterone-induced lipids accumulation and mitochondrial dysfunction

**Figure 5:**
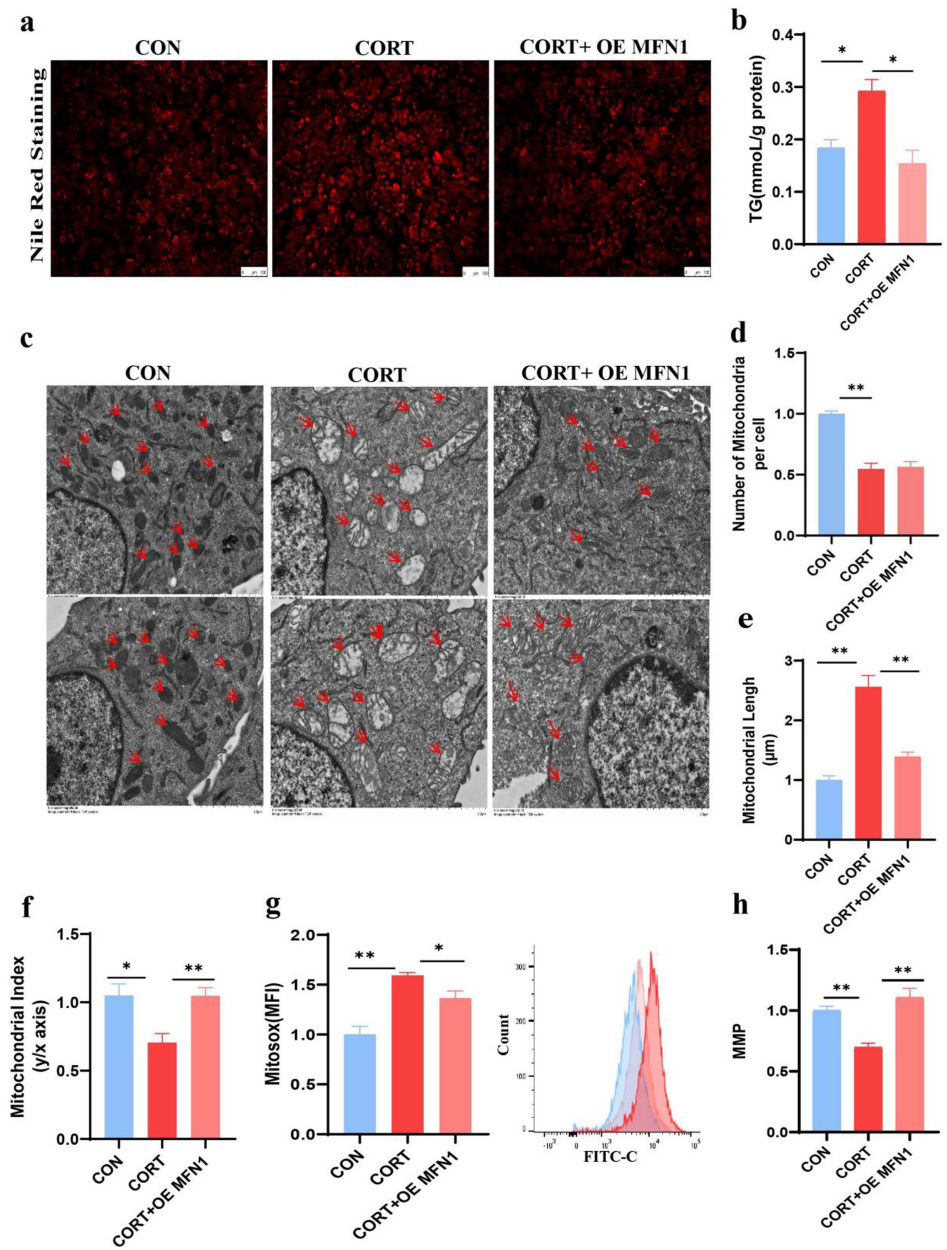
Overexpression of MFN1 alleviates corticosterone-induced lipid deposition and mitochondrial dysfunction. **(a)** Nile Red Staining of Lipid Droplet in AML12 (*n* = 3). **(b)** TG content in AML12 (*n* = 3). **(c)** Mitochondrial morphological changes (Scale bars represent 2 μm, *n* = 3). **(d–f)** Changes in mitochondrial number, length, and aspect ratio in AML12 after OE MFN1. **(g)** Mitochondrial ROS levels in AML12 (*n* = 3). (h) MMP in AML12 (*n* = 3). Values are Mean ± SEM, *p < 0.05, **p < 0.01, compared with the CORT group.

Given that MFN1 is a crucial protein for maintaining mitochondrial membrane integrity, we investigated whether restoring its expression could enhance mitochondrial function and mitigate the onset of fatty liver. Functional validation was conducted through MFN1 overexpression. Nile Red staining of AML12 demonstrated that overexpression of MFN1 alleviated lipid droplet accumulation induced by CORT (Figure 5a) and significantly reduced TG levels (Figure 5b). TEM images of AML12 mitochondria indicated that overexpression MFN1 notably restored the normal morphology and cristae structure of mitochondria, although there were no significant changes in mitochondrial numbers (Figures 5c–f). Furthermore, overexpression of MFN1 significantly improved the elevation of ROS levels and the reduction in MMP induced by CORT (Figures 5g,h). Collectively, these findings suggest that MFN1 enhances mitochondrial function by restoring mitochondrial integrity, thereby alleviating the progression of fatty liver.

### GR involves in corticosterone-induced fatty liver by regulating MFN1

**Figure 6:**
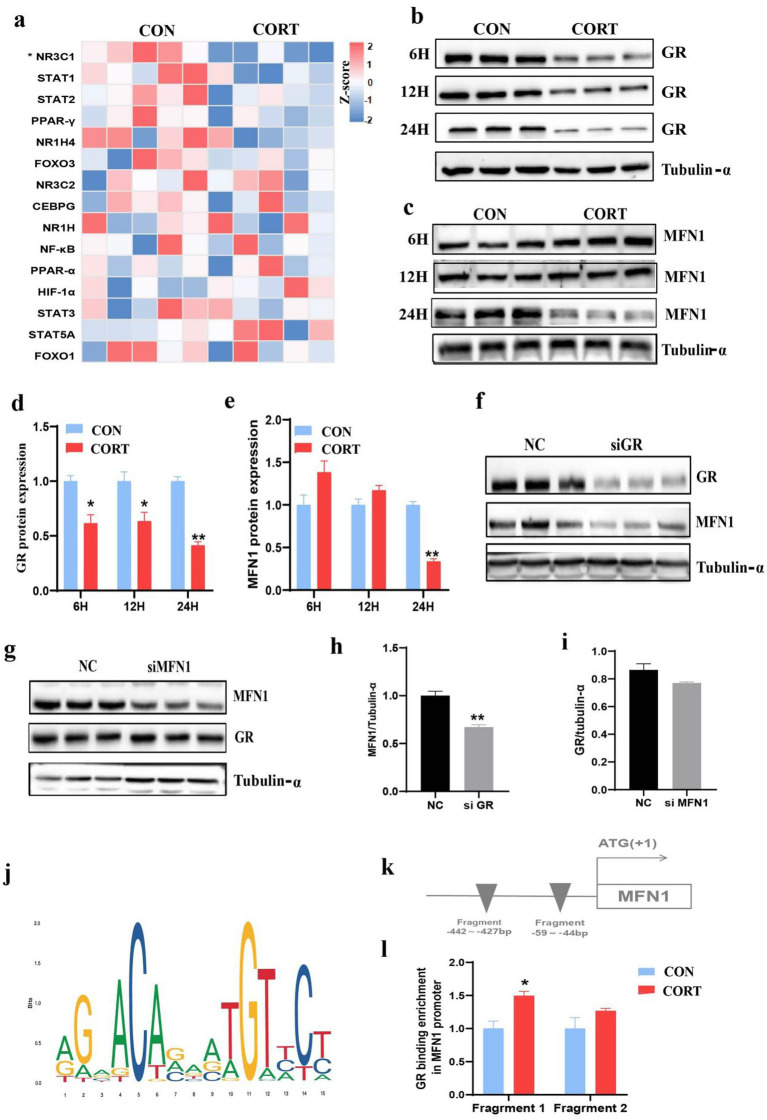
GR Involves in corticosterone-induced fatty liver by regulating MFN1. **(a)** Heatmap showing the transcriptional factors related to glucocorticoid, as measured using transcriptomics, in the CON and CORT-stimulated livers (*n* = 5). **(b–e)** GR/MFN1 expression after 6/12/24 hours of corticosterone treatment (*n* = 3). (f-i) MFN1/GR expression after GR/MFN1 knockdown (*n* = 3). **(j)** Schematic diagram of the abundance of GR binding in the initiation stage (*n* = 3). **(k)** Schematic representation of GR binding sites in the MFN1 promoter region. **(l)** ChIP-PCR analysis of GR binding to the MFN1 promoter (*n* = 3). Values are Mean ± SEM, *p < 0.05, **p < 0.01, compared with the CON group.

The functionality of glucocorticoids is depend on their receptors GR. As a transcriptional regulator, we explored the potential role of GR in CORT-induced fatty liver by modulating MFN1 expression. Heatmap showing the transcriptional factors related to glucocorticoid, only GR significantly suppressed in CORT group (Figure 6a). Given that GR function exhibits time dependence, we investigated the changes in GR expression at different time points during CORT treatment. The results indicated that GR protein expression was significantly inhibited after 6, 12, and 24 h of CORT exposure (Figures 6b,d). Additionally, since mitochondrial dynamics is a complex and dynamic process, we found that 6 and 12 h of CORT treatment had no significant effect on MFN1 expression; however, 24 h of CORT treatment resulted in a marked decrease in MFN1 protein levels (Figures 6c,e). Subsequently, we observed that knockdown of GR led to a significant decrease in MFN1 protein expression (Figures 6f,h), whereas knockdown of MFN1 did not affect GR expression (Figures 6g,i). This suggests that GR is involved in the upstream regulation of MFN1. To elucidate the mechanism underlying the changes in MFN1 expression in CORT-induced fatty liver, we utilized the JASPAR database to predict binding relationships between the mouse GR transcription factor and the *MFN1* promoter (Figure 6j). The results indicated potential binding regions of mouse GR in the *MFN1* promoter, specifically at MFN1 Fragment 1: −442 to −427 bp and MFN1 Fragment 2: −59 to −44 bp (Figure 6k). ChIP-PCR experiments revealed that the level of GR binding to the *MFN1* promoter region in the CORT group was significantly higher than that in the CON group (Figure 6l). Therefore, GR regulates the expression of MFN1 by binding to its promoter region, thereby leading to the progression of CORT-induced fatty liver.

## Discussion

In modern poultry farming, chronic stress is widely recognized as a critical factor contributing to the development of FLS. During chronic stress, the HPA (Hypothalamic-Pituitary-Adrenal) axis is activated, resulting in the release of glucocorticoids that bind to their receptors, such as the GR, to help regulate the stress response and maintain homeostasis (23). However, prolonged stress can lead to continuous activation of the HPA axis, resulting in excessive secretion of glucocorticoids, which may disrupt normal bodily functions and potentially induce disease (24). In this current study, continuous CORT treatment was employed to simulate chronic stress in broilers. Consistent with previous findings, our results indicated that chronic CORT treatment induces hepatic lipid metabolism disorders in broilers (25). Specifically, the CORT treatment led to a significant reduction in body weight alongside a marked increase in hepatic and plasma TG levels. Moreover, *in vitro* experiments have demonstrated that the disruption of hepatic lipid metabolism induced by chronic CORT treatment mainly occurs in hepatocytes. These findings underscore the critical impact of chronic stress on liver health in poultry, highlighting the importance of effectively managing stress levels in broiler production to mitigate the risks associated with FLS and related metabolic disorders.

Liver is one of the tissues with the highest mitochondrial content. As an essential site for energy conversion in the cell, mitochondria not only provide ATP but also participate in the regulation of cellular metabolism and various physiological processes (26). Therefore, mitochondrial function is a key factor in the onset and progression of fatty liver disease (27). As dynamic organelles, mitochondria adapt to fluctuations in cellular energy demands through continuous processes of fusion and fission. The integrity of mitochondrial morphology is essential for their proper function, making mitochondrial dynamics crucial for maintaining mitochondrial health. Dysregulation of these dynamics can impair mitochondrial function, ultimately affecting cellular energy metabolism and viability (28). In this study, CORT treatment induced fragmentation of liver and hepatocyte mitochondria, resulting in increased smaller mitochondria without altering the overall shape. Moreover, CORT treatment led to a significant increase in mitochondrial ROS levels and a marked decline in MMP. The above results suggest that maintaining the integrity of mitochondrial structure is crucial for its function, and any disruption to this structure may result in mitochondrial dysfunction, which can exacerbate cellular damage and lipid metabolic abnormalities. Besides, CORT treatment led to mitochondrial swelling, a decrease in mitochondrial numbers, and rupture of the inner cristae structure in AML12. These differences may be attributed to the fact that *in vitro* cell lines cannot adequately mimic normal physiological conditions *in vivo*, and there is also a difference in sensitivity to CORT between AML12 and *in vivo* cells. Future research should aim to develop more effective methods to better simulate *in vivo* conditions for verification.

Researches have shown that a disruption in mitochondrial dynamics plays a crucial role in the progression of fatty liver disease. For instance, increased mitochondrial fission can lead to an accumulation of TG within hepatocytes, while inhibiting this fission can alleviate the storage of TG in these cells (29). Studies showed that a reduction in the expression of mitochondrial fusion proteins can induce injury and lipid peroxidation in the livers of rats fed a high fat and fructose diet (30). In this study, both *in vivo* and *in vitro* results demonstrated a significant reduction in the mitochondrial outer membrane fusion protein MFN1, indicating disrupted mitochondrial dynamics. Furthermore, overexpression of MFN1 effectively restored the mitochondrial morphological integrity and functionality impaired by CORT, characterized by reduced ROS levels and stabilized membrane potential, thereby alleviating CORT-induced lipid accumulation. While mitochondrial fusion plays a vital regulatory role in the onset and progression of various diseases in mammals, reports on its significance in poultry are limited. Our study is the first to reveal the role and mechanism of MFN1-mediated mitochondrial fusion in glucocorticoid-induced fatty liver in chickens.

Studies suggest that chronic stress leads to persistent activation of the HPA axis, resulting in excessive glucocorticoid secretion that disrupts negative feedback mechanisms and decreases GR expression (31). Consistently, in this current study, transcriptomic sequencing analysis from chicken livers treated with CORT revealed a significant downregulation of GR among transcription factors related to glucocorticoids. Studies showed GR functions not only exerts biological functions as a transcription factor in the nucleus but also participates regulating the target genes in mitochondrial (32). Structural insights into glucocorticoid receptor function. Such as, GR can directly regulate target genes related to oxidative phosphorylation, which affecting the activity of the mitochondrial respiratory chain (33). GR also can regulate mitochondrial fatty acid oxidation (34). Given the time-dependent nature of GR expression and the dynamic changes in MFN1, we also examined the effects of various CORT treatment durations on both. The results indicated that GR changes before MFN1. Based on experiments with knockdown of GR and MFN1, respectively, we found that GR acts upstream of MFN1. Furthermore, predictions from a transcription factor binding site database suggested that GR could bind to the promoter region of MFN1, which was confirmed by ChIP assays. The above results indicate that GR can regulate the expression of MFN1 by binding its promoter region, which involves in the progression of fatty liver. However, the exact mechanism by which GR regulates MFN1 remains to be further explored.

## Conclusion

In conclusion, this study demonstrates that glucocorticoids disrupt mitochondrial function and promote ROS production by impairing MFN1-mediated mitochondrial fusion, which ultimately contributing to the development of fatty liver in chicken. Mechanistically, inhibition of mitochondrial fusion results from specific reduction in GR binding at the *MFN1* gene promoter region. These findings enhance our understanding of glucocorticoid-induced metabolic dysfunction and identify MFN1 as a potential therapeutic target for ameliorating oxidative stress and fatty liver disease associated with aberrant mitochondrial dynamics.

## Data Availability

The datasets presented in this study can be found in online repositories. The names of the repository/repositories and accession number(s) can be found in the article/Supplementary material.

## Glossary

ALT: Alanine aminotransferase
AST: Aspartate aminotransferase
CDS: Coding sequence
ChIP: Chromatin Immunoprecipitation
CORT: Corticosterone
Drp1: Dynamin-related protein 1
FLS: Fatty Liver Syndrome
GLU: Glucose
GR: Glucocorticoid receptor
GREs: Glucocorticoid response elements
HPA: Hypothalamic-Pituitary-Adrenal
H&E: Hematoxylin and eosin
MFN1: Mitofusin 1
MFN2: Mitofusin 2
MMP: Mitochondrial membrane potential
mtDNA: mitochondrial DNA
NASH: Non-alcoholic steatohepatitis
OPA1: Optic Atrophy 1
PA: Palmitic acid
qRT-PCR: Quantitative real-time reverse transcription polymerase chain reaction
ROS: Reactive oxygen species
siRNA: small-interfering RNA
TCH: Total cholesterol
TEM: Transmission Electron Microscopy
TG: Triglyceride
