# Supplementary material for: Chronic stress-induced downregulation of MFN1 contributes to fatty liver in chickens
Source: Front Vet Sci. 2025 Sep 25;12:1646921. doi: 10.3389/fvets.2025.1646921 (PMC12509737; doi:10.3389/fvets.2025.1646921)
Supplement: Supplementary file 1 [file Table_1.doc]

table S1. Nucleotide sequences of primers for Real-time PCR.

| gene | Primer Sequence (5’-3’) | | Used for |
| --- | --- | --- | --- |
| *MFN1* | F: ATGACAGAGGGGTCGGATGA | R: ACCAAATCGTCCCTCAGCAG | Real-time PCR |
| *MFN2* | F: CTCACTGCGAGGCCAGATAG | R: GAGGCAGACTGGGATTTGCT | Real-time PCR |
| *OPA1* | F: CCTTGCCAAACTTCTGCCTG | R: AGCTCCTATGACTTCACTAACCAA | Real-time PCR |
| *DRP1* | F: CAGCTGTTCAGGGGCTGC | R: CAGCTTCTTCACCAGCTCCA | Real-time PCR |
| *PPIA* | F: CTTCGAGCTCTTCGCTGACA | R: GCCCTTGTAGCCAAATCCCT | Real-time PCR |

**table S2.** Nucleotide sequences of primers for ChIP-PCR

| gene | Primer Sequence （5’-3’） | Usage |
| --- | --- | --- |
| *MFN1* Fragment1 | Forward TCCCTGGCAAAAGCATCTCAT  Reverse GATCGAGGGTGAAGCCCAAG | ChIP-PCR |
| *MFN1* Fragment2 | Forward CTGCCCAAAACAAACCTCGC  Reverse TATTGGCTGACGCTTCGAGA | ChIP-PCR |

**table S3**. The list of antibodies

| Antibodies | Source | Catalogue NO. | Dilution |
| --- | --- | --- | --- |
| MFN1 | Proteintech | 13798-1-AP | 1:5000 |
| MFN2 | Proteintech | 12186-1-AP | 1:8000 |
| OPA1 | ABclonal | A9833 | 1:7000 |
| DRP1 | Proteintech | 12957-1-AP | 1:5000 |
| GR | Proteintech | 24050-1-AP | 1:8000 |
| Tubulin α | Bioworld | BS1699 | 1:10000 |

**table S4**. SiRNA sequences

| Genes | sense（5'-3'） | antisense（5'-3'） | Usage |
| --- | --- | --- | --- |
| *MFN1* | GAUACUAGACACUGUGAAA | UUUCACAGUGUCUAGUAUC | Si MFN1 |
| *GR* | GUUGCAAACUCGGAAUCAA | UUGAUUCCGAGUUUGCAAC | Si GR |
